# A Mixed-Methods Study on Acceptability, Tolerability, and Substitution of Brown Rice for White Rice to Lower Blood Glucose Levels among Nigerian Adults

**DOI:** 10.3389/fnut.2017.00033

**Published:** 2017-07-20

**Authors:** Sally N. Adebamowo, Olabimpe Eseyin, Susan Yilme, David Adeyemi, Walter C. Willett, Frank B. Hu, Donna Spiegelman, Clement A. Adebamowo, The Global Nutrition Epidemiologic Transition Initiative

**Affiliations:** ^1^Department of Epidemiology and Public Health, University of Maryland School of Medicine, Baltimore, MD, United States; ^2^University of Maryland Comprehensive Cancer Center, University of Maryland School of Medicine, Baltimore, MD, United States; ^3^Office of Strategic Information and Research, Institute of Human Virology Nigeria, Abuja, Nigeria; ^4^Department of Nutrition, Harvard Chan School of Public Health, Boston, MA, United States; ^5^University of Bedfordshire Business School, Luton, United Kingdom; ^6^Clinton Health Access Initiative, Abuja, Nigeria; ^7^Department of Epidemiology, Harvard T.H. Chan School of Public Health, Boston, MA, United States; ^8^Department of Biostatistics, Harvard T.H. Chan School of Public Health, Boston, MA, United States; ^9^Department of Global Health and Population, Harvard T.H. Chan School of Public Health, Boston, MA, United States; ^10^Institute of Human Virology, University of Maryland School of Medicine, Baltimore, MD, United States

**Keywords:** brown rice, white rice, Nigeria, nutrition, glucose levels

## Abstract

**Background:**

Whole-grain products such as brown rice have been associated with lower risk of metabolic disorders including diabetes. We examined the acceptability and tolerability of substituting brown rice for white rice and the feasibility of introducing brown rice into the diet through a long-term trial to lower the risk of type 2 diabetes.

**Methods:**

Fifty-one adults residing in Abuja, Nigeria, participated in this study. Using purposeful sampling for focus group discussions (FGDs), participants were enrolled based on their age (19–25 vs. 40–60 years) and body mass index (BMI) (normal weight vs. overweight/obese). Participants tasted four meals with different constitution of brown and white rice (25:75%, 50:50%, 75:25%, and 100% brown rice). Twelve FGDs were conducted, six before and six after the food tasting. Two-hour postprandial blood glucose was measured after consumption of each rice meal.

**Results:**

The mean age of the participants was 39 (±14) years, their mean BMI was 25.6 (±5.2) and about half of them were male. Most of the participants (61%) reported that rice was their main source of carbohydrate and 67% consumed rice at least five times/week. Before the food tasting, participants considered white polished rice superior to brown rice with regard to quality, taste, and nutritional value. After the food tasting, most of the participants (49%) indicated a preference for the 100% brown rice, 19% preferred the 25% brown rice, 18% preferred the 50% brown rice, and 7% preferred the 75% brown rice meals. Factors that may affect the acceptability of brown rice include its appearance, longer cooking time, cost, limited availability, and poor appreciation of its nutritional value. In general, 2-h postprandial glucose levels were lower, after consumption of meals with higher proportion of brown rice.

**Conclusion:**

This study provides valuable insight into the acceptability of brown rice as a substitute for white rice in Nigeria. If confirmed in larger studies, these results highlight the importance of increasing awareness on the nutritional value of brown rice and support the rationale for conducting a large-scale intervention trial to examine the effect of brown rice consumption on blood sugar levels among Nigerians.

## Introduction

Globally, 415 million people had diabetes in 2015, this prevalence is expected to rise to 642 million by 2040 representing 1 in 10 adults (1). Nearly half of all adults with diabetes in Africa live in South Africa [2.3 (1.2–4.6) million], Democratic Republic of Congo [1.8 (1.5–2.2) million], Nigeria [1.6 (1.2–3.8) million], and Ethiopia [1.3 (0.8–3.5) million] (1). In Africa, at least two-thirds of the people with diabetes are undiagnosed (1).

Excess dietary energy intake is a major risk factor for type 2 diabetes (T2D) and a major contributor to the global T2D epidemic. The preference for foods that contribute to dietary energy intake has been changing in different parts of the world. In Nigeria, rice is increasingly replacing traditional bolus meals particularly in urban areas and has become the main source of dietary energy among urbanized Nigerians (2). Bolus meals are thick pastes made by adding boiling water to processed starchy vegetables or roots and consumed with a side dish of vegetables, animal proteins, and oils (2). In urban areas where incomes are highest, consumers increasingly prefer long grain, polished, and destoned imported rice, while locally milled rice is consumed mainly in the rice-producing rural areas (3).

Although Nigeria is the largest producer of rice in West Africa, it is second to China as the largest importer of rice globally (4). Most of the rice consumed in Nigeria is generally processed milled rice imported from Thailand, Brazil, India, United States, and the United Arab Emirates (4). Nigeria produced approximately 2.6 million metric tons of milled rice and imported about 3.8 million metric tons in 2014/2015 (3).

In Nigeria, locally milled rice tends to be brown having been milled just once (dehulled) and unpolished. It is characterized by significant color variation, may contain mixed varieties in a single bag, and has many stones (5). Long grain brown rice, by contrast, is imported into Nigeria, has also been dehulled but does not contain stones or chaff. Locally milled rice and imported brown rice are more nutritious compared to white rice, which has been milled several times to remove the husk, bran, and germ leaving mostly the starchy endosperm (5). Most Nigerians consume white rice on weekdays at lunch or dinnertime (2). Estimates from the West Africa Rice Development Association showed that per capita rice consumption in Nigeria nearly doubled between 1980s and 2006, growing from 15.4 to 25.4 kg/year.

Studies have shown that intake of white rice is associated with increased risk of T2D in different populations, while higher dietary intake of brown rice or substitution of brown rice for white rice in the diet may lower risk of T2D (6–8). Some intervention studies found that substituting brown rice for white rice helped reduce glycemic response (9–11). However, similar studies have not been conducted among populations in sub-Saharan African (SSA) and the acceptability of such dietary substitution in SSA remains unknown.

In this study, we examined the perceptions, acceptance, and tolerability of dietary intake of brown rice meals, barriers to substituting brown rice for white rice and the willingness of adults to participate in a long-term intervention study to examine the effect of brown rice on risk of metabolic outcomes. Additionally, we examined the impact of meals with different proportions of brown and white rice on 2-h postprandial glucose levels.

## Materials and Methods

### Study Population

We purposively enrolled non-diabetic adult Nigerians residing in Abuja, Nigeria, who were between the ages of 19–25 years and 40–60 years into this study. Because people make more independent eating decisions during early adulthood regardless of their weight (12), we selected participants in the 19–25 years age group in order to evaluate the perception and acceptability of brown rice intakes among young adults while we selected participants in the 40–60 years age group because they had higher risk of T2D, especially if they were overweight/obese. Information about the study was printed on flyers, which were distributed by hand or pasted in public areas, including grocery stores, religious centers, hotels, offices, education, and recreational centers in Abuja.

**Figure 1:**
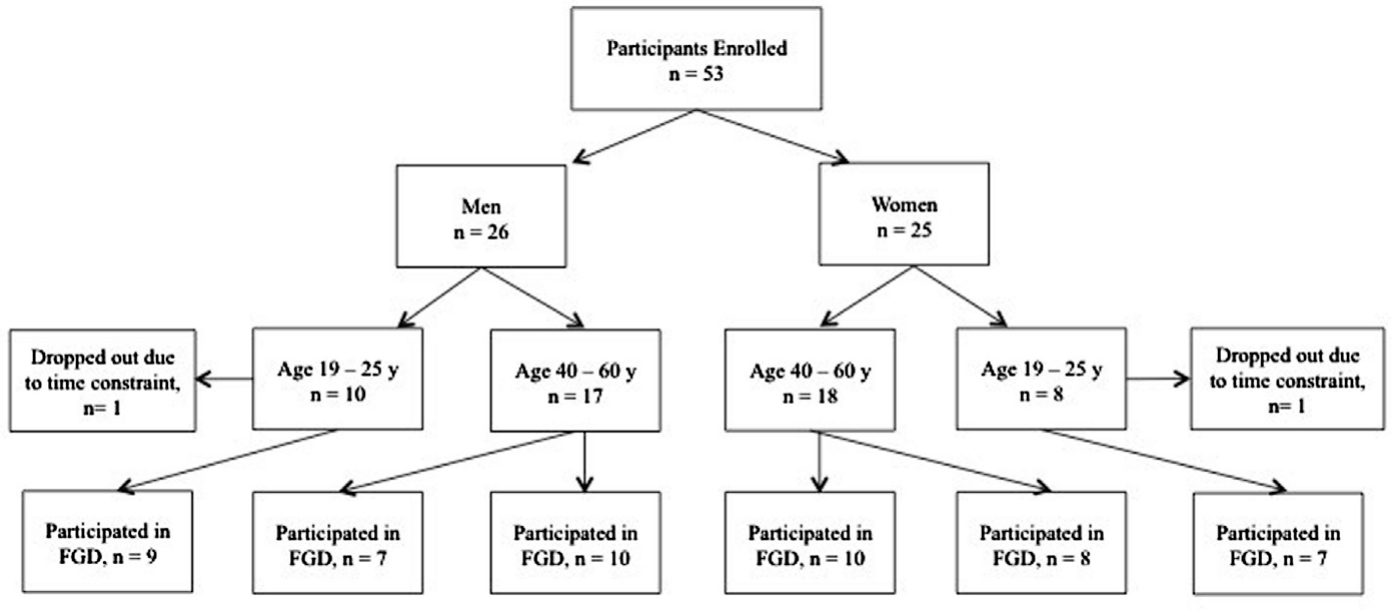
Participants distribution by gender and age.

We enrolled 53 adult Nigerians in January 2015. Two participants were unable to participate in the focus group discussions (FGDs) and food tasting parts of the study due to scheduling conflicts. The remaining 51 participants completed all the study procedures. To reduce cultural barriers to communication and encourage peer discussion, we stratified participants by age, sex, and whether they were overweight/obese or not. We, therefore, had six groups of participants composed of (1) females, aged 19–25 years (*n* = 7); (2) females, aged 40–60 years, normal weight (*n* = 10); (3) females, aged 40–60 years, overweight or obese (*n* = 8); (4) males, aged 19–25 years (*n* = 9); (5) males, aged 40–60 years, normal weight (*n* = 7); and (6) males, aged 40–60 years, overweight or obese (*n* = 10). Table 1 shows the characteristics of the study participants, and Figure 1 shows the distribution of participants by gender and age.

**Table 1:** Baseline characteristics of the study participants.

Characteristics	All, *n* (%) 51 (100)	Female, *n* (%) 25 (49)	Male, *n* (%) 26 (51)
Age^a^ (years)	39 ± 14	39 ± 12	39 ± 15
**Age groups (years)**			
19–25	16 (31)	7 (28)	9 (35)
40–60	35 (69)	18 (72)	17 (65)
Waist–hip ratio^a^	0.86 ± 0.07	0.84 ± 0.07	0.87 ± 0.07
Body mass index^a^ (kg/m^2^)	25.56 ± 5.24	24.96 ± 4.41	26.13 ± 5.96
**Body mass index (kg/m^2^)**			
Normal weight	25 (49)	13 (52)	12 (46)
Overweight	16 (31)	7 (28)	9 (35)
Obese	10 (20)	5 (20)	5 (19)
**Socioeconomic status**			
Low	21 (40)	8 (32)	11 (42)
Middle	20 (40)	14 (56)	7 (27)
High	10 (20)	3 (12)	8 (31)
**Level of education completed**			
Primary	11 (22)	2 (8)	2 (8)
Secondary	4 (8)	4 (17)	7 (27)
Tertiary	36 (70)	18 (75)	17 (65)
**Occupation**			
Unemployed	14 (27)	7 (28)	7 (27)
Self-employed	13 (26)	7 (28)	6 (23)
Skilled manual	4 (8)	1 (4)	3 (12)
Professional	20 (39)	10 (40)	10 (38)
**Marital status**			
Unmarried	21 (41)	11 (44)	10 (38)
Married	30 (59)	14 (56)	16 (62)
**Religion**			
Christian	37 (62)	19 (76)	18 (69)
Muslim	14 (38)	6 (24)	8 (31)
**Tribe**			
Hausa	6 (12)	4 (16)	2 (8)
Igbo	11 (21)	6 (24)	5 (19)
Yoruba	11 (21)	4 (16)	7 (27)
Others	23 (46)	11 (44)	12 (46)

*^a^Mean ± SD*.

### Study Procedure

On the first day of the study, all the participants were invited to the research center where they were informed about the study procedures and asked to give consent to participate in the study. After consent was obtained, data on sociodemographic profile and dietary habits were collected using interviewer-administered Nigerian food frequency questionnaires (2), height, waist, and hip circumferences were measured in accordance with the World Health Organization multinational monitoring of trends and determinants in cardiovascular disease criteria (13). Weight and body mass index (BMI) were estimated using the Omron body sensor (Omron HBF-510W Full Body Sensor Body Composition Monitor Scale) according to the manufacturer’s instructions (Omron). We conducted prefeeding FGD on the participants’ usual dietary habits, knowledge, and attitudes to consumption of different meals. Participants were then asked to return to the research center on specific days after an overnight fast (10 h), for the food tasting study. Two-hour postprandial blood glucose was measured using the Bayer’s Contour Monitoring System, according to the manufacturer’s instructions. The serial number of the batch of Bayer contour test strips we used was 301937097508.

When participants returned for the food tasting, they were served one of four different types of rice meal. We used parboiled long-grain white rice and parboiled long-grain Uncle Ben’s brown rice to prepare the meals. The first meal consisted of 25% brown rice and 75% white rice, the second had equal proportions of white and brown rice, the third had 75% brown rice and 25% white rice, and the fourth was 100% brown rice. Table 2 shows the caloric and macronutrient content of each rice meal. Each meal was assigned and referred to by a code, so participants were not aware of the composition of brown and white rice in each meal, until the codes were revealed at the end of the study. Participants were asked to use the food codes to note their perception of each meal they consumed and any symptoms such as excessive thirst, headache, mouth soreness, nausea, bloating, gassiness, borborygmi, abdominal pain, and increased stool frequency for 24 h after consumption of each meal. Each meal was served in the morning, 3 days apart, to allow ample time for participants to observe and report any gastrointestinal discomfort arising from the meals and provide a washout period between meals. One serving of chicken and tomato sauce was included with each meal. Participants were asked to consume the entire meal. The meals were consumed within 25 min, with a 500 mL drink of plain water. Two-h postprandial blood glucose was measured after consumption of each meal.

**Table 2:** Nutrient content of cooked rice meals.

Type of rice meal	Serving size (g)	Energy (kcal)	Total carbohydrate (g)	Total fat (g)	Total protein (g)	Dietary fiber (g)	Water (g)
25% brown rice and 75% white rice	300	387.0	82.1	1.5	8.9	3.3	206.6
50% brown rice and 50% white rice	300	405.0	86.1	1.8	9.0	3.9	202.1
75% brown rice and 25% white rice	300	423.0	90.0	2.2	9.1	4.5	197.6
100% brown rice	300	441.0	93.9	2.6	9.3	5.1	193.14

Organoleptic assessment of the meals was conducted using modified 10-level Likert scales, as soon as each participant completed the food tasting for each meal. We conducted postfeeding FGDs on the acceptability and tolerability of brown rice, identification of barriers to brown rice consumption, feasibility of introducing brown rice as participants’ main dietary staple, and willingness to participate in future brown rice intervention trials. The food codes were then revealed and participants were informed about the rice composition of the different meals they consumed.

### Focus Group Discussions

**Figure 2:**
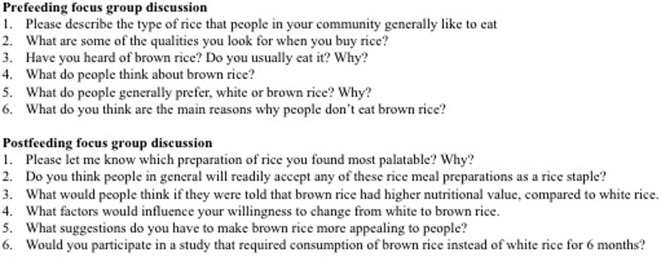
Sample questions asked during the focus group discussions.

We used A Practical Guide for Applied Research (14) to design our FGD. Members of the Global Nutrition Epidemiologic Transition Initiative in Shanghai, Dar es Salaam, and Chennai have utilized similar FGD protocols with success (15–17). Figure 2 shows a sample of the questions used to guide the FGD. In total, we conducted 12 FGDs, one for each group, about 40 min before and after eating the rice meals. Each FGD lasted about 60 min and was moderated by OE and SY, who were both graduate research associates with experience in qualitative research and DA, a physician researcher who also had qualitative research experience. All interviews were conducted in English and audiorecorded. The recordings were transcribed verbatim, ensuring there was no data loss.

### Statistical Analysis

Using grounded theory and open coding, SA analyzed the transcripts (18). Each transcript was read multiple times and coded inductively by SA and OE, using a combination of manual and computer-aided analysis. The text data were entered into ATLAS.ti^®^ software and systematically analyzed to identify recurrent themes. Descriptive analyses were used to characterize the study population using SAS 9.3 (SAS Corp., Cary, NC, USA). We used Bonferroni corrected Dunn’s test (19), Kruskal–Wallis, Wilcoxon signed-rank, χ^2^, and Fisher’s exact tests to assess differences between the groups. In order to compute SES in a low-resource environment, we generated a wealth index using the factor analysis (principal components) procedure and varimax rotation as previously described (2, 20). We determined the principal component upon which wealth index is based on the following variables: ownership of fan, bicycle, television, fridge, motorcycle, car, and home; type of residence: duplex, multiple bedroom apartment, and studio; source of drinking water: dispenser, fetch, deep well, pipe-borne, surface water, and borehole; source of cooking fuel: firewood, gas, kerosene, and electricity; type of toilet: none, water cistern, pit toilet, and aqua privy; and whether a separate room for cooking was available. Factor scores were calculated from the sum of the ownership of household items weighted by their factor loading. The data were sorted based on the first principal component, and participants were categorized as follows based on its value, the lower 40% as having low SES, the middle 40% as having middle SES, and the top 20% as having high SES. The validity and reproducibility of wealth index has been examined in previous studies, and it correlates well with other measures of wealth in environments without reliable expenditure data (20).

## Results

### Characteristics of the Study Population

There were 51 adults in this study of whom 31% (16/51) were in the 19–25 years age category and 69% (35/51) were in the 40–60 years age category. Their mean (± SD) age was 39 (±14) years; mean (±SD) BMI was 25.6 (±5.2); and mean (±SD) waist–hip ratio was 0.86 (±0.07). About half, 51% (26/51) of the participants were male, half were normal weight (49%, 25/51), some were overweight (29%, 15/51), and some were obese (22%, 11/51). Most of the participants had completed university education (70%, 36/51) and were employed (73%, 37/51). Some of the participants were married (59%, 30/51).

### Attitude to Rice Intake

Most of the study population (61%, 31/51) reported that white rice was their main source of carbohydrate. It was consumed more frequently on weekdays compared to weekends. The reported frequency of white rice consumption was 14% (7/51) for more than once daily, 22% (11/51) for 5–7 times per week, 47% (24/51) for 2–4 times per week, and 17% (9/51) for once per week or less frequently (Table 3).

**Table 3:** Dietary characteristics of the study participants.

Characteristics	All, *n* (%) 51 (100)	Female, *n* (%) 25 (49)	Male, *n* (%) 26 (51)
**Main source of carbohydrate**			
Rice	31 (61)	17 (68)	14 (54)
Fufu (swallows)	9 (17)	4 (16)	5 (19)
Yam	7 (14)	3 (12)	4 (15)
Bread	2 (4)	1 (4)	1 (4)
Others	2 (4)	0 (0)	2 (8)
**Frequency of white rice consumption**			
>/1 day	7 (14)	5 (20)	2 (8)
5–7/week	11 (22)	9 (36)	2 (8)
2–4/week	24 (47)	8 (32)	16 (61)
≤/1 week	9 (17)	3 (12)	6 (23)
**When rice is usually consumed**			
Weekdays	32 (63)	15 (60)	17 (65)
Weekends	19 (37)	10 (40)	9 (35)
**Ever eaten brown rice**			
Yes	4 (8)	3 (12)	1 (4)
No	47 (92)	22 (88)	25 (96)

*Fufu (swallows) are bolus meals made by adding boiling water to processed starchy vegetables or roots to form a thick paste*.

During the prefeeding FGDs, participants reported a preference for imported white long-grain rice, compared to local unpolished rice because the former was usually more readily available in the market, less expensive, and did not contain chaff or stones. A participant said:

“In my place, they prefer the whiter rice because it does not involve stress of picking stones like the local one.”

Participants indicated that consumption of white polished rice was associated with affluence and assumed it was more nutritious because it was imported.

“I eat white rice because if you have white rice at home you are likely to be rich.”

Even those who ate local unpolished rice at home would buy white polished rice as gifts for their friends and family.

“If you are coming from the town and you feel you want to buy something for your parents, you could buy the foreign rice.”

### Awareness and Perceptions of Brown Rice

Most of the study participants had no prior knowledge of brown rice. They said:

“I’ve only heard of one type of rice, it is not brown.”

“I’ve never seen or heard of brown rice.”

Some participants who had heard of brown rice thought it was a meal for sick people. Comments included:

“I have heard [of it] and eaten it before, they call it diabetes rice.”

“I heard it is used for people with kwashiorkor.”

“I have seen it but not eaten it [brown rice], I heard it is medicinal.”

Some participants thought that *tuwo shinkafa*, a local bolus meal made from rice flour, was similar to brown rice. Only 8% (4/51) of the participants had ever eaten brown rice before this study, all of whom reported that they liked it a lot. Many participants expressed belief that brown rice had low nutritional value.

“I don’t think the brown rice has any nutritional value.”

### Organoleptic Assessment

In general, the 25% and 50% brown rice meals had the best organoleptic ratings. Participants rated the appearance and color of the 100% brown rice lower than the other meals. For elasticity, there was a significant difference between the 75% brown rice/25% white rice meal, compared to the 100% brown rice meal. The acceptability of all meals was high, and there was no significant difference between the different rice meals. Table 4 shows the organoleptic assessment of each meal.

**Table 4:** Comparison between organoleptic assessment of 100% brown rice to other rice meals.

Organoleptic characteristics	100% brown rice	25% white rice and 75% brown rice	*p*-Value^a^	50% white rice and 50% brown rice	*p*-Value^b^	75% white rice and 25% brown rice	*p*-Value^c^
Appearance	7.5 ± 2.1	7.8 ± 1.8	0.86	7.9 ± 1.8	0.06	7.5 ± 1.9	0.21
Color	7.3 ± 2.4	7.6 ± 1.9	0.47	7.6 ± 2.0	0.16	7.4 ± 2.2	0.14
Luster	7.6 ± 2.2	7.6 ± 2.0	0.36	7.6 ± 2.3	0.71	7.4 ± 2.1	0.84
Shape	7.6 ± 2.0	7.7 ± 1.9	0.43	7.6 ± 1.9	0.52	7.6 ± 2.1	0.98
Aroma	7.9 ± 1.8	7.8 ± 1.8	0.97	8.1 ± 1.7	0.48	7.9 ± 2.2	0.60
Flavor	8.2 ± 1.8	8.3 ± 1.7	0.27	8.4 ± 1.8	0.86	7.9 ± 2.0	0.82
Texture	8.0 ± 2.1	8.0 ± 1.8	0.06	7.8 ± 2.0	0.79	7.6 ± 1.7	0.91
Taste	8.5 ± 1.7	8.5 ± 1.6	0.70	8.7 ± 1.5	0.36	8.5 ± 1.7	0.71
Rigidity	7.9 ± 2.0	7.7 ± 1.9	0.12	8.1 ± 1.6	0.55	7.5 ± 1.8	0.74
Elasticity	7.8 ± 2.2	7.5 ± 2.3	0.009	7.9 ± 2.0	0.56	7.3 ± 2.3	0.34
Overall acceptability	9.1 ± 1.9	9.0 ± 2.1	0.90	9.3 ± 1.3	0.58	8.8 ± 2.2	0.54

*Wilcoxon signed-rank test was used to assess the mean difference between 100% brown rice and the other meals*.

*Values are mean organoleptic score ± SD*.

*^a^100% brown rice meal vs. 25% white rice/75% brown rice meal*.

*^b^100% brown rice meal vs. 50% white rice/50% brown rice meal*.

*^c^100% brown rice meal vs. 75% white rice/25% brown rice meal*.

### Tolerability of the Rice Meals

Some participants (23%, 12/51) reported symptoms of ill health after eating the rice meals. Of these, seven were 19–25 years old and five were 40–60 years old; eight were male and four were female; and nine were normal weight and three were overweight/obese. These 12 participants reported 6 different types of illnesses. Two participants reported abdominal pain after eating two different rice meals, one after eating 25% and 50% brown rice meals while the other reported abdominal pain after eating 25% and 100% brown rice meals. A third participant reported abdominal pain after eating three different rice meals with 25%, 50%, and 75% brown rice but not 100% brown rice meal. The other symptoms reported by participants included diarrhea (*n* = 3), thirst (*n* = 2), and gassiness (*n* = 1) after eating the 25% brown rice meal; diarrhea (n = 5), thirst (*n* = 2), gassiness (*n* = 2), headache (*n* = 2), and abdominal pain (*n* = 1) after eating the 50% brown rice meal; diarrhea (*n* = 1), thirst (*n* = 1), and borborygmi (*n* = 1) after eating the 75% brown rice meal; and thirst (n = 1) and gassiness (n = 1) after eating the 100% brown rice meal.

### Dietary Preference after the Food Tasting

In the postfeeding FGD, most participants (49%, 25/51) reported a preference for the 100% brown rice meal. Fewer participants (19%, 10/51) indicated a preference for the 25% brown rice meal, 18% (9/51) for the 50% brown rice meal, and 14% (7/51) for the 75% brown rice meal. Table 5 shows the participants’ postfeeding meal preference by BMI categories. Participants reported that their preferences were related to the taste, texture, color, and aroma of the meals. Some comments from the postfeeding FGD included:

“The taste [of the 100% brown rice meal] was okay but I can’t say much about its color.”

“I preferred the 50% white rice and 50% brown rice because it was not too hard and not too soft, compared to the 100% brown rice, which was a little bit hard.”

“I didn’t like the 25% brown rice, the aroma was not good.”

**Table 5:** Participants postfeeding meal preference.

Type of rice meal	Total, *n* = 51	Normal weight, *n* = 25	Overweight, *n* = 15	Obese, *n* = 11

	*n* (%)			
25% brown rice and 75% white rice	10 (19)	7 (28)	3 (20)	–
50% brown rice and 50% white rice	9 (18)	3 (12)	4 (27)	2 (18)
75% brown rice and 25% white rice	7 (14)	3 (12)	2 (13)	2 (18)
100% brown rice	25 (49)	12 (48)	6 (40)	7 (64)

### Acceptability of the Rice Meals

The most common barriers to the acceptance of brown rice reported by the participants in this study were lack of awareness of its nutritional value, longer cooking time, cost, and limited availability. A participant said:

“If it is available everywhere, that will help.”

Some participants were concerned that consumption of brown rice may be considered a symbol of lower living standards. The participants agreed that to promote acceptability of brown rice, effort should be focused on creating awareness, educating the public on its nutritional value, and increasing its availability. The appearance of brown rice was also discussed as an important factor that may hinder its acceptability. Comments included:

“Do something to improve the appearance. We have polished rice and the other one [local rice], this brown rice can be made brighter.”

“It is not shining in appearance.”

“The appearance will be the problem if I serve it to guests during an occasion.”

Advertising was mentioned as an important tool that could promote awareness. Others mentioned that if there was no significant difference between the price of brown and white rice, more people would consume it. Other suggestions included introducing brown rice to children so that they become accustomed to it at an early age and creating new brown rice recipes to improve its taste.

### Participating in Long-term Intervention Studies

Most participants (88%, 44/51) indicated willingness to participate in 6 months long brown rice intervention trials designed to examine the effect of daily consumption of brown rice on cardiometabolic risk. Those who indicated they were unlikely to participate in such trials mentioned time constraint as a major disincentive.

### Blood Glucose Tests

**Figure 3:**
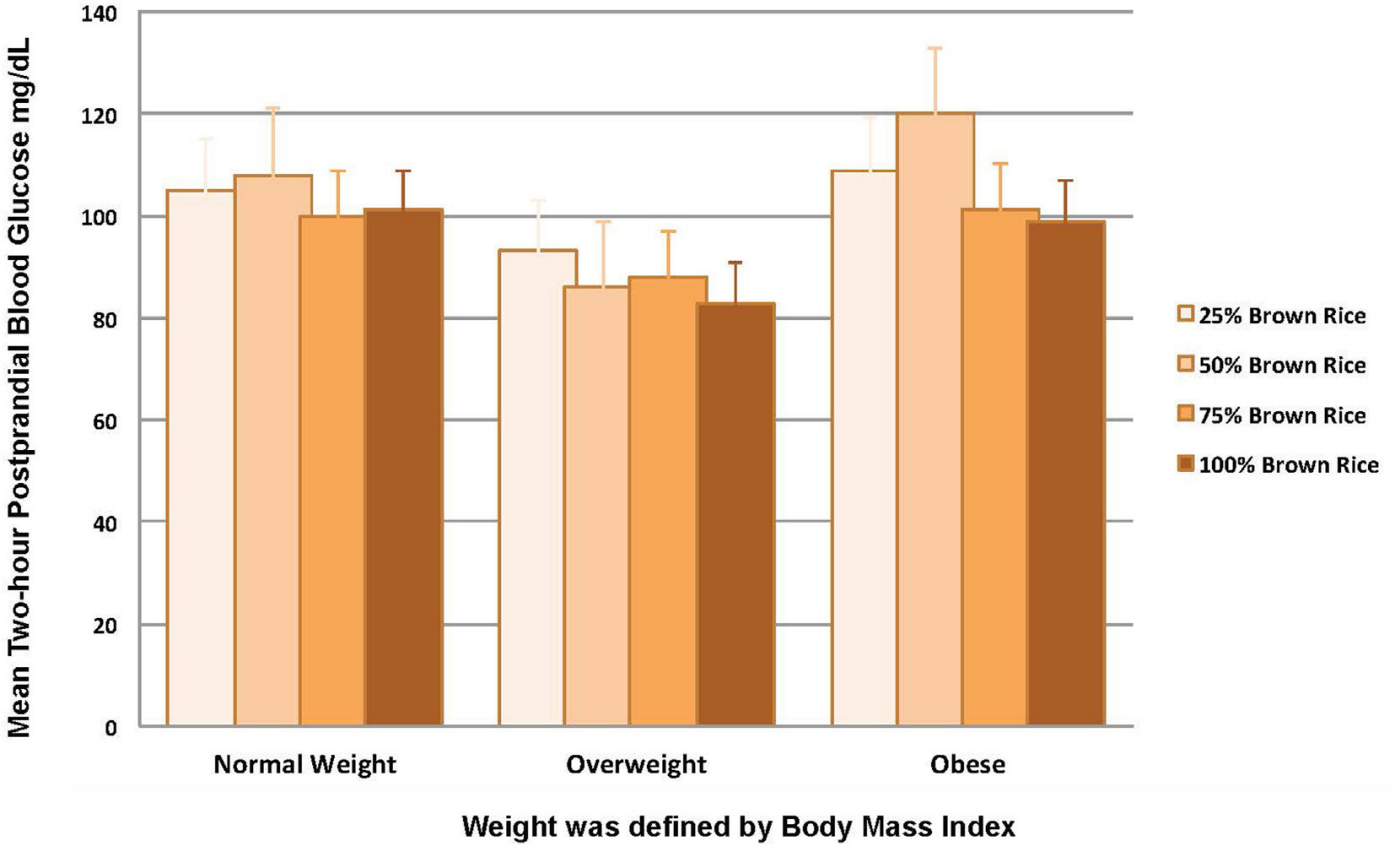
Two-hour postprandial blood glucose levels for each rice meal.

The participants mean (SD) 2-h postprandial blood glucose was 102 (63) mg/dL after eating the 25% brown rice meal, 104 (65) mg/dL after eating the 50% brown rice meal, 97 (49) mg/dL after eating the 75% brown rice meal, and 96 (38) mg/dL after eating the 100% brown rice meal. We observed that in general, 2-h postprandial glucose levels were lower, after consumption of meals with higher proportion of brown rice (Figure 3). In analyses stratified by BMI, we observed a statistical difference in the distribution of 2-h postprandial blood glucose, comparing normal weight to obese participants, after consumption of the 25% brown rice/75% white rice meal (Bonferroni-corrected *p* value 0.04), the 50% brown rice/50% white rice meal (Bonferroni-corrected *p* value 0.002), the 75% brown rice/25% white rice meal (Bonferroni-corrected *p* value 0.004), but not the 100% brown rice meal (Bonferroni-corrected *p* value 0.29), Table 6. We also observed a statistical difference in the distribution of 2-h postprandial blood glucose, comparing overweight to obese participants, after consumption of the 50% brown rice/50% white rice meal (Bonferroni-corrected *p* value 0.005), the 75% brown rice/25% white rice meal (Bonferroni-corrected *p* value 0.03), the 100% brown rice meal (Bonferroni-corrected *p* value 0.03), but not the 25% brown rice/75% white rice meal (Bonferroni-corrected *p* value 0.38), Table 6.

**Table 6:** Multiple comparisons of mean 2-h postprandial glucose for the rice meals.

	25% brown rice/75% white rice		Kruskal–Wallis *p*-value*
	Normal weight	Overweight	
Overweight	0.40		0.07
Obese	0.04	0.38	

	**50% brown rice/50% white rice**		

	Normal weight	Overweight	
Overweight	0.98		0.003
Obese	0.002	0.005	

	**75% brown rice/25% white rice**		

	Normal weight	Overweight	
Overweight	0.86		0.01
Obese	0.004	0.03	

	**100% brown rice**		

	Normal weight	Overweight	
Overweight	0.24		0.06
Obese	0.29	0.03	

*p-Values were estimated with the Dunn’s test for multiple comparisons with Bonferroni adjustment*.

*Kruskal–Wallis p-values, comparing all BMI categories.

## Discussion

This study on substituting brown for white rice identified barriers that affect the acceptability of brown rice and factors that may enhance acceptability of brown rice as a staple food in Nigeria. Less than 10% of the participants had ever eaten brown rice before the study. After the food tasting, most participants indicated a preference for the 100% brown rice meal, compared to other meals with lower proportion of brown rice (25%, 50%, and 75%). The distribution of 2-h postprandial blood glucose was different after consumption of meals with 25%, 50%, and 75% brown rice, comparing normal weight to obese participants, and after consumption of meals with 50%, 75%, and 100% brown rice, comparing overweight to obese participants. Among overweight or obese individuals, we observed that in general, the mean 2-h postprandial glucose levels were lower after consumption of meals with higher proportions of brown rice.

In this study, we observed that rice was the main source of carbohydrate intake, replacing traditional bolus meals, which is consistent with the result of our previous study conducted in 2010 among urbanized adult Nigerians (2). Most rice intake is on weekdays reversing the trend from largely weekend consumption in the past. About four decades ago, imported long grain parboiled rice was consumed at weekends, celebrations, and parties by most Nigerians, whereas consumption of locally grown rice was more prevalent, albeit not as frequently as bolus meals (2). In this study, we observed a preference for imported white long grain parboiled rice over local rice. This may be related to the wide availability and packaging—clean, free of stones and chaff—of imported white rice.

Awareness of the existence and nutritional value of brown rice in Nigeria is poor. The general perception of brown rice is that it had lower nutritional value compared to white rice. Paradoxically, people believed that brown rice is a better option for individuals who were ill, because of its nutrient content. Creating awareness and educating the people are important steps toward changing the attitude of Nigerians toward brown rice. Introducing brown rice to children, adolescents, and young adults before they establish their dietary habits and incorporating it in menus at local restaurants will boost its acceptability. Using brown rice instead of white rice in popular local rice recipes, such as fried or other mixed rice dishes, will mask its color and encourage more people to try it without being deterred by its appearance. Currently, a pack of white rice (10 kg) costs about 10,000 Naira, compared to a similar pack of brown rice which costs 1,500 Naira in Nigeria. People may be more inclined to try brown rice if it was available in small affordable packages per serving size in local markets. Wider availability of parboiled brown rice, which will save on cooking time, may help make brown rice more acceptable to individuals with busy schedules. Given that most participants did not report any symptoms of ill health after the food tasting study, tolerability of brown rice may not affect its acceptability, even among individuals who have never eaten brown rice.

Results from observational studies have shown that substitution of whole grains, including brown rice, for white rice may lower risk of T2D (7, 8). A crossover design trial conducted among adults randomly allocated to daily consumption of white rice, brown rice, or brown rice with legumes for five consecutive days found that substituting brown rice for white rice may be associated with reduced 24-h glucose and fasting insulin response among 15 overweight and obese adults (11). Although the methodology was different from ours, these findings support the hypothesis of lower 2-h postprandial blood glucose levels after consumption of brown rice compared to white rice. Diets that include brown rice have also been shown to be associated with lower blood glucose levels among adults with impaired fasting glucose or T2D (9). It has been suggested that the insoluble fiber in brown rice is responsible for lowering the postprandial blood glucose concentration (21). However, the results from intervention studies have been inconsistent (9, 10, 22, 23). A study conducted among 202 adults found that incorporating brown rice into the daily diet for 16 weeks did not substantially improve blood glucose levels (22). Other short-term intervention trials found some (9, 10) or no (10, 23) benefit for substituting whole grain for refined grains on metabolic risk. These results suggest that intervention studies with larger sample sizes and longer duration of follow-up should be conducted to ascertain the impact of brown rice intakes on glucose metabolism. Most of the participants in our study were willing to participate in such long-term trials.

Our study has several strengths and limitations. We used a qualitative study approach, which enabled an evaluation of individuals’ knowledge, perception, and attitude within homogenized groups, in a relaxed setting. During the food tasting, study participants were blinded to the rice composition of the meals, which allowed an unbiased assessment of the preference and tolerability of each meal. Our study was limited to adults, so our results may not be generalized to children and adolescents. As we did not control for all potential confounding factors, we cannot rule out the possibility of residual confounding in our study. Given the small sample size and cross-sectional design, our study was not adequately powered to evaluate the impact of brown rice intakes on glucose metabolism and we were unable to rule out other factors that may have contributed to the symptoms experienced after consumption of each meal.

In conclusion, our study identified the perception and factors that may hinder the acceptability of brown rice among adult Nigerians. Awareness of brown rice is low in this population and there are misconceptions about its nutritional value. After tasting brown rice, most of the participants indicated preference for it and expressed willingness to participate in long-term brown rice intervention trials. Brown rice consumption may be associated with lower 2-h postprandial blood glucose levels. If confirmed by large-scale studies, these findings may guide policies and the market structure in the rice sector to stimulate availability and affordability of brown rice in Nigeria.

## Ethics Statement

The study was approved by National Health Research Ethics Committee of Nigeria. All the study participants were informed about the study and required to sign informed consent forms before participation.

## Author Contributions

SA developed the study proposal and protocol, conducted the analysis, depicted the data, wrote the manuscript, provided significant intellectual interpretation for the discussion of all the results, and was primarily responsible for the completion of the manuscript. OE, SY, and DA contributed to the study protocol, coordinated the study, and compiled the data. WW, FH, DS, and CA contributed significant ideas to the manuscript’s structure, discussion, and summary. CA obtained funding to conduct the study. All the authors read and approved the final manuscript.

## Conflict of Interest Statement

The authors declare that the research was conducted in the absence of any commercial or financial relationships that could be construed as a potential conflict of interest. The reviewer, AC, and handling editor declared their shared affiliation, and the handling editor states that the process nevertheless met the standards of a fair and objective review. The reviewer, MU, declared a shared affiliation, though no other collaboration, with two of the authors, SA and CA, to the handling editor, who ensured that the process nevertheless met the standards of a fair and objective review.
